# The Effectiveness of Inquiry and Practice During Project Design Courses at a Technology University

**DOI:** 10.3389/fpsyg.2022.859164

**Published:** 2022-05-18

**Authors:** Jing-Yun Fan, Jian-Hong Ye

**Affiliations:** ^1^Department of Fashion Design, Tainan University of Technology, Tainan, Taiwan; ^2^Faculty of Education, Beijing Normal University, Beijing, China

**Keywords:** curriculum confidence, curriculum interest, curriculum value perception, fashion design, inquiry and practice, inquiry-based learning, project design, time series analysis

## Abstract

Among the many teaching methods, inquiry-based teaching is considered to be an effective way for students to learn and solve problems on their own. However, most of the research related to inquiry-based teaching and learning has concentrated mainly on K-12 education, while few to no studies have focused on the application of inquiry-based teaching and learning in project design courses at university level. Therefore, in order to expand the understanding of the application effect of inquiry-based teaching at university level, this study adopted the quasi-experimental design method, and through the purposive sampling method, 20 students from the Department of Fashion Design at a University of Science and Technology were invited to participate in this study. During the 9-month period, teaching experiments were carried out using two inquiry models, QC/ADEAC and QD/ODEAC. First, when participants were thinking of a creative topic, they followed the process: Question (Q), Collection/Analysis (C/A), Discussion (D), Explanation (E), Amendment (A), and Confirmation (C) in the course. During the production process, the participants were allowed to improve on their work through the process of Question (Q), Doing/Observation (D/O), Discussion (D), Explanation (E), Amendment (A), and Confirmation (C). The teacher became a true guide, so that the participants could explore and work out how to improve their designs through independent inquiry and practice. In this study, questionnaires were administered to participants at five important stages of the design project: “theme development,” “color development,” “first Work,” “second Work,” and “third Work.” The results of the five surveys showed that the participants’ curriculum interest, curriculum value perception, and curriculum confidence in the inquiry program all increased.

## Introduction

In project design, students use a design and planning process to investigate a core problem, and learning occurs when students engage in a largely self-directed process that involves finding, coordinating, and selecting a functional solution to the problem (Hong et al., 2012). This is because students must be involved in the in-depth problem-solving process, which triggers progressive inquiry learning through the practical experience of solving contradictory problems (Muukkonen et al., 2005). In inquiry-based learning, the teacher’s role is to guide students, help them practice critical thinking, enhance their higher-level thinking and problem-solving skills, guide them to understand scientific concepts, and enhance their knowledge of the role of science process skills and their motivation to learn (Tseng et al., 2013).

Therefore, inquiry-based learning is considered an active learning method that has been shown to be beneficial for developing students’ inquiry skills, and for improving their academic performance, engagement, and motivation (Kori, 2021). At the same time, inquiry-based learning helps learners develop the ability to work in complex and unpredictable environments, enabling them to become more critical thinkers and active learners (Suarez et al., 2018). Despite the benefits of using inquiry-based learning, it is still relatively uncommon for higher education institutions to use it compared to other educational levels (Kori, 2021).

Inquiry-based learning can best occur through project implementation (Wilhelm et al., 2008). Another concept that can be integrated with inquiry-based learning is topic-based learning (Kori, 2021). When people encounter a new topic, they try to understand it while also participating in it and looking back at what they know or need to know about the project in order to complete the topic (Hong et al., 2011). In topic-oriented learning, learners are usually more concerned with the final product, and the learning process follows more steps (Kori, 2021).

The purpose of inquiry-based learning is to engage students in the real process of scientific evidence, explicitly from a pedagogical perspective, by dividing the complex scientific process into small but logically connected units of learning, thereby drawing students’ attention to the important details of scientific thinking (Pedaste et al., 2015). The main goal of classroom-based inquiry learning is to allow students to design their own experiments, collect data from the experiments, interpret the results, justify the conclusions with the results, and communicate the results of the experiments to others (Teig et al., 2018). In other words, inquiry-based learning is an approach that allows students to conduct their own scientific experiments to construct knowledge, rather than acquiring new knowledge directly from the teacher’s lecture process (Jerrim et al., 2020), which shows that inquiry-based learning is a student-centered teaching method with many scientific evidence-based steps. However, the literature does not precisely define how inquiry-based learning is implemented, and learning models take many forms; inquiry-based learning is also related to other different learning strategies that include many modern teaching practices (Cairns and Areepattamannil, 2019).

Analysis of the literature on inquiry-based learning revealed that it is associated with many different learning and teaching strategies, including many teaching methods and types that are relevant to modern teaching practices, and for which different models and step-by-step processes can be found (Cairns and Areepattamannil, 2019; Kori, 2021). For example, a typical implementation of inquiry-based learning is through a semester-long course project (Mountrakis and Triantakonstantis, 2012). Project-based learning is a closely related application of inquiry-based learning in which students must complete research projects, and past experience in teaching project-based learning has taught instructors two things. First, students cannot be left alone, but need active support and regular feedback; second, students need a clear structure and curriculum to orient their learning methods and tasks (Mieg, 2019).

This echoes Murphy et al.’s (2021) view that inquiry-based learning is defined in a variety of ways, but is often interspersed with terms such as hands-on activities, student-centered practices, and problem-based or topic-based learning. It is clear that project-based inquiry-based learning can help students define their learning methods and tasks, and engage in inquiry-based learning in a progressive manner. Therefore, this study adopted an inquiry-based learning approach based on the project. According to Tretter and Jones (2003), the implementation of inquiry-based learning can take different forms depending on classroom conditions. In essence, inquiry-based learning is the application of laboratory work to classroom learning. Therefore, in this study, we developed inquiry models applicable to project design in project design courses based on a circular model, and further explored the learning effects of their use.

Learning is motivating, and the more students believe they have learned, the higher their interest in participating in learning activities (Bulunuz et al., 2012). Thus, interest has many positive effects on learning processes and outcomes (Krapp, 2002), this is thought that interest may have an inspirational effect in guiding students to choose certain goals or in helping them to pursue them (Hui and Bao, 2013). Therefore, interest in learning is also considered as a motivation for learning (Ye et al., 2020), and a strong interest in learning activities is considered as an essential condition for learning (Hong et al., 2017). Dewey suggests that stimulating learners’ interest is a better way to teach than forcing learners to take learning seriously (Chen et al., 2016). In addition, affective factors also influence students’ motivation and effectiveness (Sara and Maria, 2013; Baars et al., 2017; Eckerlein et al., 2019), so understanding students’ interest in inquiry-based learning courses will help to understand their motivation to learn, so this study considers interest in courses (affective factors) as one of the variables to be explored.

Perceived value refers to how important, interesting, and enjoyable students perceive an activity, as well as a subject or terminology (Mills and Moulton, 2017), so superficial learning strategies occur when students’ perceived value of the curriculum is low (Floyd et al., 2009), and students’ choice of learning environment may be influenced by students’ perceptions of learning comfort (Clayton et al., 2018). Students’ choice of learning environment may be influenced by the perceived value of the curriculum in terms of comfort (Clayton et al., 2018). Therefore, it is considered necessary to take into account students’ perceived value when modifying the teaching process, since an increase in perceived value will increase students’ propensity to continue learning (Dlačić et al., 2014).

Inquiry-based learning is considered a meaningful approach to learning, but it is more important to understand students’ ideas about their interest in the curriculum and values, which are concepts or beliefs that are cognitive reflections of universal human needs (Schwartz, 1994), and values are ideas, principles, and rules that guide people’s lives and that people accept, prefer, and consider important for judging and evaluating actions (Knafo and Schwartz, 2004). Therefore, understanding students’ perceived value of the inquiry-based learning curriculum will help to understand their recognition of the curriculum, so this study considers curriculum value (cognitive factors) as one of the variables to be explored.

Furthermore, self-confidence is a person’s belief that he or she can succeed in the context of a particular task (Perry, 2011), and is considered an important component in the context of learner-centered learning and curriculum reform (Maclellan, 2014). Therefore, students’ confidence in completing academic tasks can be a key factor in understanding student achievement behaviors and academic outcomes (Liem et al., 2008). This may allow students to believe in their own abilities, not to give up easily even when they encounter problems, to complete all tasks independently, and to gain the greatest sense of achievement (Safitri and Widjajanti, 2019). Whereas confidence may decrease depending on the content, topic, or situation being discussed (Perry, 2011). Students’ self-confidence can be maximized through cognitive approaches (Liyadipita, 2021). Therefore, understanding students’ self-confidence in the inquiry-based learning curriculum will help to understand their beliefs about their ability to participate in the curriculum, so this study includes self-confidence in the curriculum (cognitive factors) as one of the variables to be investigated.

Based on the above, the results of this study will help faculties in design departments understand how to effectively implement inquiry-based teaching methods in their courses. At the same time a time-series analysis questionnaire was also used to understand how learners’ cognitive and affective perceptions changed during the implementation of this innovative teaching method. Based on the aforementioned research purposes, the research questions for this study are as follows:

Research Question 1: What are the application processes of “QC/ADEAC inquiry model” and “QD/ODEAC inquiry model” in the project design course?

Research Question 2: How effective is the application of inquiry teaching method in the project design course of University of Science and Technology?

## Literature Review

### Inquiry-Based Learning

Inquiry generally refers to a range of educational approaches with some key common features, including a curriculum that actively considers students’ interests, and students asking questions and conducting authentic, discipline-based, or interdisciplinary individual or group extension units (Borovay et al., 2019). During the investigation phase, students identify issues related to the problem by designing the investigation, conducting experiments, and interpreting and evaluating the results (Teig et al., 2018). Interpretation involves thinking, feeling, and reacting in ways that are both cognitive and emotional, providing a way to determine how individuals experience the world and make sense of those experiences (Hong et al., 2011). Therefore, this is another important process of inquiry in addition to exploring the problem.

Hong et al. (2019) found different descriptions of the inquiry models presented by different researchers using various terms to mark very similar stages. However, it is important to design an inquiry model that can be simplified to actually be conceptually independent. Inquiry-based learning is often organized into inquiry phases that together form an inquiry cycle (Pedaste et al., 2015). After Hong et al. (2021) reviewed all of the inquiry-based learning stages, they concluded that different inquiry models are applicable to different types of learning content. For example, Yang et al. (2021) proposed the QODE model based on an online inquiry-based learning approach which includes Questions (Q), Observation (O), Doing (D), and Explanation (E), and other processes. Fan and Ye (2022) proposed the QC/ADEAC model based on an online inquiry-based learning approach which includes Questions (Q), Collection/Analysis (C/A), Discussion (D), Explanation (E), Amendment (A), and Confirmation (C). Hong et al. (2019) proposed a POQE model based on the characteristics of the course platform, including Prediction (P), Observation (O), Quiz (Q), Explanation (E), and other processes. In addition, Hong et al. (2020) also proposed a PD/OQ/DE/T model based on a generic inquiry-based learning approach which consists of a Prediction (P)—Doing/Observation (D/O)—Question/Discussion (Q/D)—Explanation/Transfer (E/T) process. The investigation model developed in this research will be based on the above-mentioned investigation model.

### Curriculum Interest

Interest has been conceptualized as the mental state of engaging with specific types of objects, events, or ideas over time (Hidi and Renninger, 2006) and as the person-object relationship that leads to engagement or re-engagement, and on topics related to abstraction and knowledge-based learning. Learner interest has been identified as a key indicator that facilitates motivational processes (Schrader et al., 2021). The necessary condition for learning is that students develop a strong interest in the learning activity (Hong et al., 2017). Therefore, whether students have a strong interest in the inquiry and hands-on curriculum is one of the indicators of successful teaching of the new curriculum approach. Based on this concept, this study examined students’ perceived interest in the curriculum when they participated in inquiry-based learning.

### Curriculum Value Perception

Value assessment is the subjective value students place on a learning activity, outcome, or domain (Pekrun et al., 2011). Therefore, students’ evaluation is an important variable in promoting learning (Özgüngör, 2010), so when modifying the teaching process, tertiary institutions need to consider students’ perceived value, and an increase in perceived value also increases the propensity to continue learning (Dlačić et al., 2014). Therefore, for the inquiry and practice courses that belong to innovative teaching, understanding the value of the course as perceived by students will help to expand the understanding of the benefits of the course. Students’ perceptions of the value of the curriculum when they participate in inquiry-based learning were measured based on this concept.

### Curriculum Confidence

Confidence is an individual’s belief in his or her own ability to predict future performance (Hong et al., 2017). Maclellan (2014) suggested that confidence is a component of each individual’s self-expression that plays a role in a range of performance indicators and is related to the impression, content, or situation of an individual’s competence in a particular domain. Confidence is an emotional construct which implies that a person who knows what to do and how to do it believes that he/she can accomplish tasks and maintain positive outcome expectations (Stajkovic, 2006). Therefore, participants’ confidence in inquiry and practical work will affect their success in inquiry-based learning. Therefore, based on this concept, this study examined students’ perceptions of their confidence in participating in inquiry-based learning in the curriculum.

## Research Design

### Method

For ethical or budget reasons, random assignment in teaching practice is usually not feasible, and when randomized experiments are not feasible, a quasi-experimental design approach can be used instead to evaluate the experimental effects (Kim and Steiner, 2016). In the quasi-experimental design, the one-group time-series analysis of the single-group quasi-experimental design has multiple measurement time points in the whole experimental process. The researcher inserted an experimental treatment at one of these time points to observe changes in the subjects at each time point, which is thought to be particularly applicable to evaluating ongoing instructional programs in schools (Johnson, 1986). In addition, the single-group quasi-experimental design does not reduce its validity compared to the two-group design (Yin, 2003). Thus, the use of single-group quasi-experimental design in teaching experiments is an effective research method to help solve the problem of having too few participants or not being able to implement traditional experimental designs. Therefore, this study adopted the quasi-experimental design method of a single group, and the teaching experiment lasted for 9 months. In order to understand the cognitive and emotional state of participating in the inquiry-based learning course, this study adopted the self-report method of the participants filling out questionnaires, to see their perceptions of the inquiry-based learning curriculum during the 9-month teaching experiment.

### Teaching Model

The creative design process can be described as an iterative and cyclical process in which the different stages—conceptualization, experimentation, reflection, and revision—are mixed and repeated, and through which students find design ideas and develop these ideas until the desired design creation is realized (Seitamaa-Hakkarainen et al., 2001). The creative design process is similar in many ways to a step-by-step inquiry-based process. Therefore, this study explores the development of the model by combining the creative design steps proposed by Seitamaa-Hakkarainen and the following related literature.

Hong et al. (2019, 2020), Yang et al. (2021), and Fan and Ye (2022) proposed inquiry processes echo Tretter and Jones’ (2003) and Murphy et al.’s (2021) suggestion that inquiry-based experiences present different steps and approaches depending on classroom conditions. However, what remains unchanged is that in the inquiry-based learning method, the teacher always plays the role of supporting learning, while students actively build their knowledge base through the process of searching for information, asking questions, and finding answers; the inquiry-based learning process therefore also includes the process of collecting and analyzing data (Chu et al., 2011). Aditomo et al. (2013) argued that inquiry-based learning is knowledge construction driven by questions, which means that students must conduct some kind of investigation in order to solve the problem, and this concept is the basis of the analysis step. The analysis step is crucial for project design, so this process step was included in the investigation model developed in this study.

In addition, Xenofontos et al. (2020) indicated that the core components of the inquiry cycle are carefully choreographed because the stages and steps have interrelated concepts. Hsiao et al. (2017) also proposed an inquiry model design based on a circular inquiry model that could help students construct knowledge through repetitive steps. Therefore, in this study, the design of the inquiry history was based on the cyclic inquiry model. In addition, the QODE inquiry model of Yang et al. (2021) and the QC/ADEAC inquiry model for thematic development in design departments of Fan and Ye (2022) were used as the basis for the development of the inquiry model. This study combined the above concepts and was guided by three experts with backgrounds in both technical and vocational education and design education to develop the “QD/ODEAC inquiry model” for practical application and verify the two models. In addition, since the QC/ADEAC and QD/ODEAC model of inquiry, the former focuses on design ideas while the latter focuses on practical work, and the implementation steps are slightly different, meanwhile this study applied both of them.

**FIGURE 1:**
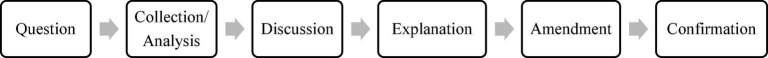
The QC/ADEAC inquiry model.

The steps of the “QC/ADEAC inquiry model” applied to the development of the topic include: Question (Q)—students explore what direction they want to design and create based on the theme of the graduation exhibition; Collection/Analysis (C/A)—students perform data collection and analysis based on the relationship between the creative questions and the theme of the graduation exhibition; Discussion (D)—students explain and discuss their ideas with the instructor based on the results of their data analysis; Explanation (E)—the instructor explains the analysis errors or imperfections of the problem in the results of the student inquiries, that is, the relevant design principles implied in the development; Amendment (A)—students reanalyze the errors or imperfections according to the feedback from the instructor; Confirmation (C)—after students have made corrections, they confirm to the instructor that the corrections are correct, as shown in Figure 1.

**FIGURE 2:**
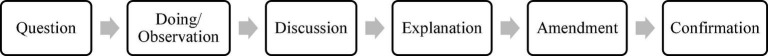
The QD/ODEAC inquiry model.

The QD/ODEAC inquiry model for practical work consists of: Question (Q)—students explore what process they would like to use based on their own design direction; Doing/Observation (D/O)—students conduct practical work on different craft techniques and observe whether the best effect is presented by the technique they want to use; Discussion (D)—students and the instructor discuss the results of their practical work and observations; Explanation (E)—the instructor provides feedback on the rationality of the design based on the content of the students’ explanations and suggests ways to improve the quality of the work; Amendment (A)—students rework their works based on feedback from the instructor to address errors or imperfections; Confirmation (C)—students correct the work and confirm to the instructor that the corrections are correct, as shown in Figure 2.

### Teaching Implementation

While executing project design, students may find and solve many problems. In this approach to learning, students are usually asked to complete a task and must investigate and explore the causes behind the problem themselves to produce a complete design. Therefore, at the beginning of the semester, the study first announced the concept of this inquiry-based learning curriculum (i.e., students are the main actors of independent knowledge inquiry and teachers are the supporting actors of knowledge absorption) according to the principle that students were free to choose to participate in this study’s inquiry-based learning design project curriculum, which was designed to motivate more students to join by gaining a sense of commitment from students and teachers. In addition, the study also addressed the issue of attendance and teaching practices by keeping the number of participants within a manageable range.

The project design program was designed to guide students in the production of fashion accessories such as bags, shoes, and boots for their graduation exhibitions. During the summer and fall semesters, 36 weeks (9 months), the researcher conducts inquiry and practical courses in the project design program at the school where the teacher teaches, with two 100-min sessions per week.

This course mainly used the QC/ADEAC and QD/ODEAC inquiry models to guide students through the complete project design process from theme, inspiration (including developing imagery and color schemes to match the theme), personal creativity and style (including drawing design drafts, developing plates, finding suitable materials, making models, and finished products), and presentation (displaying exhibition boards, design drafts, models and finished products).

### Participants

The participants in this study were 20 students from the Department of Fashion Design at a University of Science and Technology in southern Taiwan, including four male and 16 female students. In this study, the gender imbalance is due to the fact that the majority of students in fashion design majors in Chinese communities are female.

### Measurement

This study collected data through a questionnaire, which was adapted from previous research instruments, and was subjected to a two-round content validity review by three experts with expertise in inquiry-based learning to confirm the integrity of the questionnaire content and the comprehensibility of the text. The Likert 5-point scale (1–5 for *strongly disagree* to *strongly agree*) was used to measure students’ perceived curriculum interest, value, and confidence in participating in the course through inquiry and practice.

#### Curriculum Interest

This study modified Hong et al.’s (2014) interest scale with nine items to measure participants’ perceived interest in an inquiry-based and hands-on thematic design course. An example item is: “I find this way of teaching very interesting.” The Cronbach’s alpha value for this scale was 0.81, the CR value was 0.85, the factor loading values ranged from 0.67 to 0.98, and the AVE value was 0.66.

#### Curriculum Value Perception

This study referred to and modified Nguyen et al.’s (2021) activity value scale with five items to measure participants’ perceptions of the value of the curriculum based on inquiry and hands-on design of the project. An example item is: “Attending this course has enhanced my ability to expand my thinking and inferencing.” The Cronbach’s alpha value for this scale was 0.83, the CR value was 0.88, the factor loading values ranged from 0.76 to 0.80, and the AVE value was 0.61.

#### Curriculum Confidence

This study revised Hong et al.’s (2017) inquiry-based learning confidence enhancement scale with six items to measure participants’ perceived confidence in the curriculum of the topic design after participating in inquiry-based and hands-on work. An example item is: “Designing projects through inquiry-based learning has given me more confidence in understanding the content of the projects.” The Cronbach’s α value for this scale was 0.97, the CR value was 0.98, the factor loading values ranged from 0.95 to 0.96, and the AVE value was 0.91.

## Results

### Reliability Analysis

In this study, the internal consistency of the scale was confirmed by Cronbach’s α. Hair et al. (2019) suggested that Cronbach’s α should be higher than 0.70 to meet the acceptable standard. The Cronbach’s α values in this study ranged from 0.94 to 0.98, which met the recommended standard, as shown in Table 1.

**TABLE 1:** Reliability analysis.

Constructs	*M*	*SD*	Cronbach’s α
Curriculum interest	4.36	0.66	0.97
Curriculum value perception	4.37	0.64	0.94
Curriculum confidence	4.28	0.72	0.98

### Time Series Analysis

**FIGURE 3:**
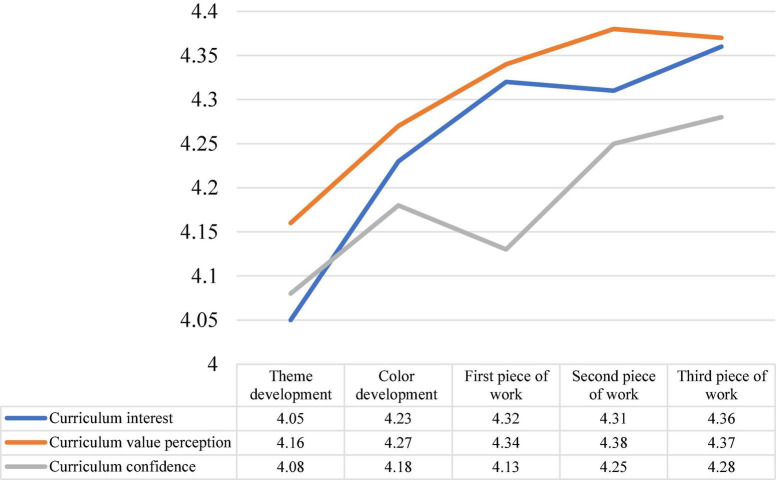
Time series analysis.

A time-series analysis was used to examine the learners’ affective performance in theme development (first survey; course month 2.5), color development (second survey; course month 3.5), first piece of work (third survey; course month 5.5), second piece of work (fourth survey; course month 7), and third piece of work (fifth survey; course month 9). In all five surveys, the participants’ perceptions of the three dimensions remained above 4 (satisfied), which means that they maintained positive feelings toward the course throughout the 9 months. The fifth survey on learners’ perceived interest in the course increased by 0.31 on average compared to the first survey; the fifth survey on perceived value of the course increased by 0.21 on average compared to the first survey; and the fifth survey on perceived confidence in the course increased by 0.20 on average compared to the first survey, as shown in Figure 3.

### Project Design Template

The project was designed in five stages. The QC/ADEAC inquiry model was used in the two stages of “theme development” and “color development.” The QD/ODEAC inquiry model was used in these three phases of the implementation of “first piece of work,” “second piece of work” and “third piece of work.” However, due to the limitation of space, only one student (hereinafter referred to as Student A) is listed as an example in this study. In addition, due to space limitation, this study only used theme development to demonstrate the QC/ADEAC inquiry model and did not repeat the color development example.

#### Theme Development of the QC/ADEAC Inquiry Model

In the first 10 weeks of the first phase of this study, the QC/ADEAC inquiry model was used to develop student themes in six stages. In the first stage, “Question,” students were given a design creation question and were asked to confirm their design theme at the end of the sixth stage, “Confirmation.” In this study, the QC/ADEAC inquiry model of the students in this course was summarized and analyzed step by step.

##### Question

**FIGURE 4:**
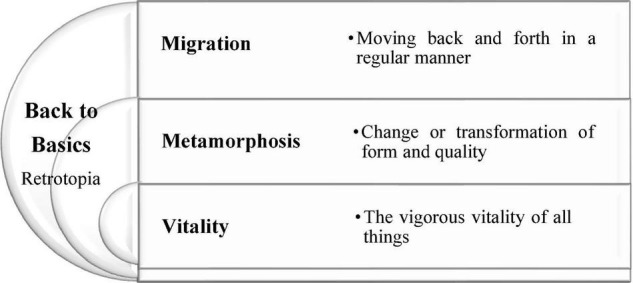
Major theme and sub-theme association diagram and meaning.

After the first 3 weeks of course development and student voting, the major theme of the graduation exhibition was determined as “Back to Basics, BB,” and the three sub-themes were “Migration, MGN,” “Metamorphosis, MTPH,” and “Vitality, VTL,” with Figure 4 as its association map and meaning. In the first step of inquiry-based teaching, “Question” refers to “How should students develop their own creative themes based on the major theme?” The first step of the inquiry-based instruction is “How should students develop their own creative themes based on the major theme?”

##### Collection/Analysis

**FIGURE 5:**
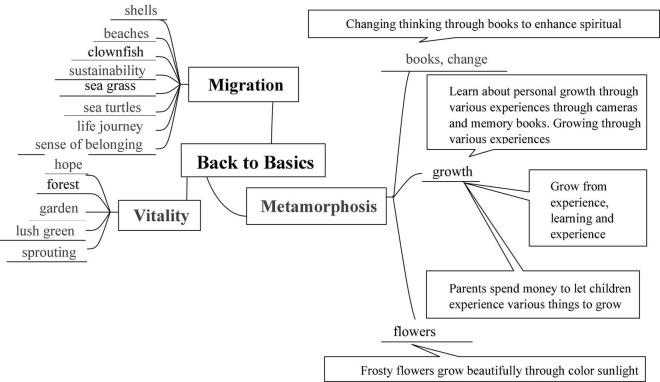
A mind map for the development themes.

In order to develop a personal theme that fits the major theme and the three sub-themes of the Graduation Exhibition for the Class of 2022, students first collected data online using documentary analysis, searched for inspiration, conducted idea searching and idea generation, and then explored their personal themes by using the mind mapping method, using “key words” and “radioactive structures” to draw a mind map by hand. The XMind 8 Update 9 software was used to create a mind map of the development of Student A’s theme, which can be seen in Figure 5.

##### Discussion

Student A used the data from the “Collection/Analysis” to develop a mind map of her theme, analyze the relevant literature (including text and graphics). The researcher asked Student A to present the collected data. Student A was asked to think about how to organize and summarize these materials. She was then asked to re-examine her thematic developmental mind map, from which she found the most data representing the sub-theme of “metamorphosis” and wanted to “*combine it with the image of frosty flowers melting in winter to show the different characteristics of human beings after growing up.*” In this stage of the inquiry process, Student A continued to analyze and interpret the data and wrote down her initial decision actions based on the key words needed for the development of her theme.

##### Explanation

**FIGURE 6:**
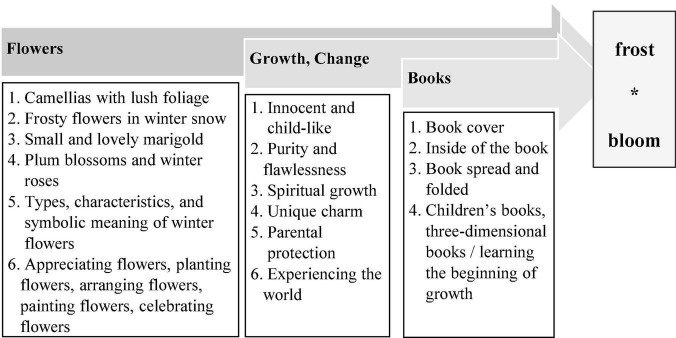
Flow chart for the development of student A’s initial theme.

At the end of each week, students were asked to write a report on the “project design inquiry and practical course learning record report” designed by the institute and upload it to the university’s online platform. The researcher reviewed Student A’s learning record after the previous lesson and found that she wrote down the key words required for the development of the theme “growth, change, books, flowers,” and initially developed the theme “frost * bloom.” The flowchart and related words, phrases, and sentences are shown in Figure 6.

##### Amendment

**FIGURE 7:**
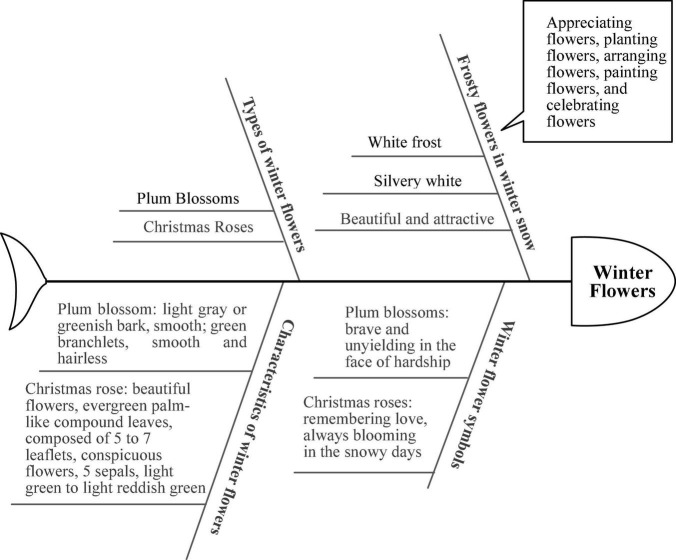
Student A’s correction theme of the fishbone diagram.

Student A used “Winter Flowers,” the most popular theme in the previous phase of development, as a concept revision theme and hand-drew a fishbone diagram to develop a thematic network focusing on winter flowers. I We used the XMind 8 Update 9 software to draw the fishbone diagram of student A’s correction theme, as shown in Figure 7. We found that the student focused the thematic development on the concept of “winter flowers.” Therefore, it was recommended that she explore and develop the theme directly from this.

##### Confirmation

The researcher reviewed Student A’s “project design inquiry and practical course learning record report” on the theme of exploring and revising the content related to “winter flowers,” which mentioned that:

“*Through the gradual receding of the frost and snow that covers the plum blossoms in late winter and early spring after being nourished by sunlight, we compare the process of human growth and metamorphosis, and transform it into a creative inspiration for our works.*”

Student A also took the symbolic meaning and shape of plum blossoms and presented her bag creation in a way that “the emotion is in the scene,” using both flat and three-dimensional compositions to create a “*late winter and early spring, when the sunlight penetrates the sky, and the frost and snow recede as the weather warms up, and the flowers gradually reveal their colors.*” As a result, student A decided on the theme of “Winter Flowers.” In addition, after deciding on this theme in week 10, Student A continued to search for imagery, develop a color scheme, and draw a design based on the theme, all following the “QC/ADEAC inquiry model.”

#### The QD/ODEAC Inquiry Model of Work Production

In the first 18 weeks, Student A developed their themes in the “QC/ADEAC inquiry model” and decided on the theme of their graduation exhibition, “Winter Flowers,” then searched for imagery, developed color schemes, drew designs, and finalized the project. In the following 18 weeks, the first work, the second work, and the third work were developed in the “QD/ODEAC inquiry model” based on the finalized design, and the three works for the solo exhibition were finalized. The following is a step-by-step analysis of the development and production of Student A’s first work using the “QD/ODEAC inquiry model.”

##### Question

Student A confirmed the theme of her graduation exhibition as “Winter Flowers,” and the imagery of the theme was interpreted as “*The flowers in the late winter gradually bloom with their own unique colors under the nourishment of warm light, just as people change their thinking through learning, knowledge brought by education and books, experience and physical transformation, and reveal their different qualities.*” The first step of the “QD/ODEAC inquiry model,” “Question,” is “*How can I integrate the concept of book opening and closing into bag design and production?*” The first step in the QD/ODEAC inquiry model was “How can I integrate the concept of book opening and closing into bag design and production?”

##### Doing/Observation

At this stage, student A first drew the design, during the drawing process, and were required to refer to different data and paper models or prototype production, and compare their characteristics with the combination of the results, in order to confirm the most suitable design or production results. In addition, student A used the Adobe Illustrator vector graphics software to simulate the first piece of artwork based on the final draft of the design of “Winter Flowers,” as shown in Table 2.

**TABLE 2:** Student A’s final draft design, “winter flowers.”

Item	Front view	Side view	Overhead view	After closing
Design draft	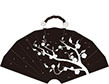	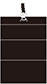	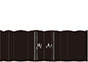	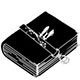

The mock-up process must consider the bag opening structure, function, material, hardware, manufacturing method, and so on, and constantly observe and compare the various considerations that bag professionals will make before producing the finished product.

##### Discussion

Student A used Adobe Illustrator vector graphics software to simulate the first piece of work, in order to consider all aspects of the design before it is produced by bag professionals. Student A discussed with the researcher from design to pattern making, the physical properties of leather and other materials in relation to pattern making, the correlation between the thickness of leather and pattern making, the comprehensive analysis of processing techniques, the correlation between hand stitching or sewing stitches and needle adjustment, the choice of machine, and the correlation between the structure of leather parts and the type of sewing machine.

The researcher asked student A to define the width, depth, and height of the bag according to the design, and to think about the structure of the pattern before making the layout. It took a long time to make the plates, and the teacher kept checking and giving appropriate suggestions during the process. Student A mainly used the computer to open the plates and output each plate, and then tried to make paper molds with white newsprint. In this process student A repeated the cycle of “open plates→make paper molds→correct the plates→correct the paper molds” and kept observing and comparing whether the paper molds made for the bag corresponded to the design draft and had the function of opening and closing like a book. Table 3 shows the paper bag mold for Student A’s “Winter Flowers.”

**TABLE 3:** Student A’s “winter flowers” paper bag mold.

Front view	Side view	Overhead view
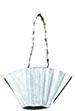	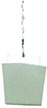	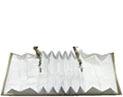

*Size: Width (W.) × Depth (D.) × Height (H.) = 21 cm × 68 cm × 35 cm Strap Drop = 20 cm.*

##### Explanation

Student A completed the paper model of the bag in the previous stage, and the exhibition plate and paper model were examined for the first time. The judges gave the following suggestions.

“*The shape is original, the structure is quite creative, the material is not yet defined and the structure is reasonable.*”

The process of exploring the design to the pattern and paper mold in the previous stage helped Student A choose the thickness and type of leather, hardware, and other sub-materials, and then confirm the processing technology of this bag. In the “project design inquiry and practical course learning record report” feedback, the student noted:

“*Because the teacher’s advice gave me a direction to look in, I also discovered many materials that I hadn’t used before, and the material cost a lot of money.*”

##### Amendment

Student A first encountered many difficulties in the development of the plate, for example, the original manual development, but could not accurately produce the book opening and closing functions, and because of the complex structure of the bag, changed to a computer development plate. Next, she chose cowhide as the main material to make the front and back pieces of the bag, but because cowhide is not easy to shape, it could not create the effect of a book opening and closing, and so she changed to using washed kraft paper.

Student A gave the following feedback in the “project design inquiry and practice course learning record report”:

“*The production process was difficult, but fortunately I didn’t give up, because I didn’t need to consider the inside when making the paper mold, and I added the inside to the finished product, so I thought a lot about “how to make the inside” during the process.*”

After completing the first product and going through the third review, Student A discussed with the instructor to revise the product in response to the reviewers’ suggestions.

##### Confirmation

Student A’s finished product is shown in Table 4. In addition, she wrote down her creation experience in the “project design inquiry and practical course learning record report”:

**TABLE 4:** The first finished product of “winter flowers” by student A.

Front view	Side view	Overhead view
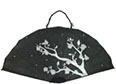	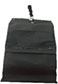	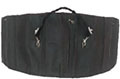

*Size: Width (W.) × Depth (D.) × Height (H.) = 21 cm × 68 cm × 35 cm Strap Drop = 20 cm.*

“*The work was created by observing the surrounding things. Due to the complex layout, it was mainly carried out in the way of computer printing. When making the work, I encountered difficulties again and again, and the process of solving them one by one constantly improved the details. Various bag-making techniques were used to make the work appear.*”

### Examples of the Work

In this project, each student was asked to design a series of three pieces of artwork based on the major theme of the graduation exhibition, and they were judged by internal and external design experts. Twenty students who participated in this inquiry-based teaching program passed the review. In project design, students need to apply higher order cognition, a neurodevelopmental function consisting of a complex set of thinking skills including concept acquisition, systematic decision making, evaluative thinking, brainstorming (including creativity), and rule use (Levine, 2009), in order to produce creative work. In addition, teachers who encourage active discussion among students during lessons can enhance students’ higher levels of cognition (Dubey and Dubey, 2017). The students in this study used verbal and non-verbal language to describe or reflect on concepts, choose appropriate strategies and think flexibly to solve problems in the design process, and generate ideas through brainstorming to complete their personal work. Therefore, in this study, these works, which were reviewed and approved by internal and external experts, were considered as a cognitive expression of inquiry-based learning. A sample of related works is shown in Table 5.

**TABLE 5:** Examples of the work.

No.	Front view	Side view
1		
	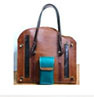	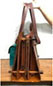
2		
	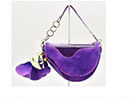	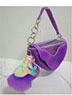
3		
	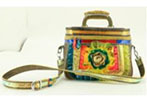	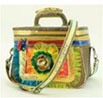
4		
	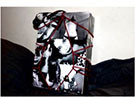	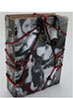
5		
	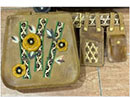	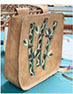
6		
	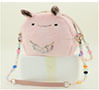	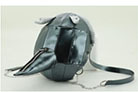

### Discussion

#### Research Question 1: What Are the Application Processes of “QC/ADEAC Inquiry Model” and “QD/ODEAC Inquiry Model” in the Project Design Course?

Pedaste et al. (2015) suggested that from a pedagogical perspective, complex scientific processes that are divided into subtler and logically connected steps can guide and draw students’ attention to important features of scientific thinking. Therefore, the step-by-step inquiry model can help students to conduct an in-depth scientific inquiry. In addition, because design creation requires continuous experimentation to find the best and most appropriate effect, the design process also involves repetition. Thus, in this study, the QC/ADEAC inquiry model and the QD/ODEAC inquiry model were designed for the two major stages of thematic development and practical work of thematic design, which are effective in the “inquiry-based” design creation process. The two models adopted in this study were also consistent with Cairns’ (2019) argument that the active, more self-directed, and student-centered nature of inquiry-based learning allows students to be participants in knowledge generation rather than being passive recipients of knowledge. It also implicitly engages students in the scientific investigative skills that are prerequisites of equipment use, data generation and processing, and reasoning (Cairns, 2019). In summary, this study identified a model of inquiry that is applicable to each project design phase at a University of Science and Technology.

#### Research Question 2: How Effective Is the Application of Inquiry Teaching Method in the Project Design Course of University of Science and Technology?

Inquiry teaching can produce positive cognitive and affective outcomes (Borovay et al., 2019). Therefore, in this study, questionnaires were administered to participants at five important stages of the design project: “theme development,” “color development,” “first Work,” “second Work,” and “third Work.” The results of the five surveys showed that the participants’ curriculum interest, curriculum value perception, and curriculum confidence in the inquiry program all increased. In the last survey, the participants’ perceptions of curriculum interest, value, and confidence in inquiry were all greater than 4.0, which mean that the participants recognized the positive learning benefits of the inquiry model in the project design curriculum.

This is in line with previous literature such as Hong et al. (2017), who stated that a necessary condition for learning requires a strong interest in the learning activity. When the brain makes a connection between the activity that elicits interest and positive feelings, this makes us want to repeat the behavior (Bressler et al., 2021). However, interest can, over time, engage specific types of objects, events, or mental states of ideas (Hidi and Renninger, 2006). A great interest in learning can contribute to success in a variety of domains (Zheng, 2021).

Mansour (2015) noted that the value of inquiry-based learning for student learning and engagement in science classrooms is increasingly recognized. Value assessment is considered to be the subjective value that students place on a learning activity, outcome, or domain (Pekrun et al., 2011), so when learners perceive a higher level of value, it indicates a higher level of recognition of the curriculum activity. The results of this study showed a high level of perceived curriculum value, which means that participants recognize the project based on the inquiry and practical model.

Moreover, confidence is considered as various beliefs of someone that recognizes his/her ability and competence in a particular academic subject (Sheldrake, 2016). Therefore, confidence should be considered as a student’s quality in which the student believes that he/she has the confidence to successfully complete different activities inside and outside the classroom in order to achieve the learning objectives. Confidence then affects the success of students’ learning process and outcomes (Kapur, 2008).

In addition, this study pointed out the influences of confidence in the learning process, which refers to how students’ learning changes when they experience high or low confidence (Akbari and Sahibzada, 2020). As shown in Figure 3, the participants in this study had consistently high levels of confidence in the inquiry and practical project design course.

## Conclusion and Recommendations

### Conclusion

Although problem-based learning and topic-based learning are often used in the design field at universities of technology, the concept of “inquiry-based” is less commonly mentioned and few studies have been conducted on it. The development of an inquiry-based approach to the design field will facilitate the implementation of inquiry-based teaching in this field. Therefore, this study developed and verified the inquiry models based on the concept of cyclic inquiry learning. When students made memory or comprehension errors in the project design, they could use this approach to provide immediate feedback which helped them to correct themselves. The development of an inquiry teaching model applicable to the design field is also one of the contributions of this study. On the other hand, the results of the analysis showed that the students were positive about this learning model and it helped them to improve their confidence in inquiry. It also indicated that they had a deeper understanding of the QDEAC science concepts and knowledge, and gradually improved their learning effectiveness in project design (design creation) through the inquiry-based activities they participated in.

Newton and Tonelli (2020) suggested that inquiry based on a practical curriculum is a way for students to find their own answers, correct misconceptions from mistakes, and then share what went wrong and how to improve. While inquiry-based learning is the ideal learning principle for higher education, it depends on students’ motivation and independence to achieve self-constructed knowledge. Therefore, in order to maximize students’ knowledge inquiry, the instructor played the role of a facilitator throughout the 9-month teaching period to help students construct their own knowledge.

During the implementation of this study, we found that the “QC/ADEAC inquiry model” for theme development and the “QD/ODEAC inquiry model” for artwork production can develop students’ abilities of “problem identification,” “planning and research,” “argumentation and modeling,” and “presentation and sharing.” For example, in the “Question” and “Collection/Analysis” stages of theme development, students developed their own theme for their graduation exhibitions based on the major theme, and had to observe social phenomena, collect information on popular trends, and so on. Through the process of collecting information, reading, and discussing, students proposed appropriate design inquiry questions and came up with their own ideas for their graduation exhibition themes.

In the “Question” and “Doing/Observation” stage of the work production, students used the Adobe Illustrator vector graphics software to simulate their first work by considering the bag opening structure, function, material, hardware, production method, and so on, and constantly observed and compared the various considerations that bag professionals make before producing the finished product. The students were able to observe and compare the various considerations that bag professionals make before they produce the finished product, which cultivated the students’ ability to “find problems.”

In the “Collection/Analysis” stage of theme development, students used the appropriate tool of mind mapping to draw up a personal theme development plan, collect data continuously, design an appropriate recording format and record each idea in detail, and to plan appropriate methods, materials, and processes according to the questions raised by the teacher. In the “Doing/Observation” stage, students were required to work on different techniques and observe whether the best results were achieved by the techniques they wanted to use. In the “Discussion,” “Explanation,” and “Amendment” stages of theme development, students analyzed and interpreted data, created a flow chart of theme development based on the collated data, collected data systematically with a fishbone diagram, examined the most appropriate conditions, proposed conclusions or solutions based on them, and finally developed and established their own graduation theme. In the “Discussion,” “Explanation,” and “Amendment” stages, students used the Adobe Illustrator vector graphics software to simulate their first work, develop a layout, make a paper model, and finally complete the final product.

During the “Discussion,” “Explanation,” “Amendment,” and “Confirmation” stages of theme development and production, students presented their inquiry results in a structured manner, used various resources to share information with others, used symbols or models to present their inquiry process and results, and used oral, written, and multimedia expressions appropriately to present their inquiry process and results. In the process, they collaborated and discussed with their peers, listened to others’ reports, provided specific comments to evaluate their peers’ inquiry processes, results, or models, and proposed reasonable improvement plans. In addition, in the fifth stage, “Amendment,” and the sixth stage, “Confirmation,” students proposed ideas or models based on the selected questions, evaluated and judged various types of information, and critically examined their applications.

### Recommendations

Summarizing the above findings and conclusions, this study recommends the following.

#### The Instructor Needs to Adopt a Variety of Strategies to Enhance Learners’ Ability to Deconstruct the Problem

Inquiry-based learning is defined as instruction that presents students with a specific challenge, through which they can learn to acquire a great deal of information, record their personal learning, and explore the problem by setting up a path to find a solution. However, when students encounter problems during the implementation process, they must have the basic knowledge, concepts, and abilities to overcome and complete the tasks. Although much information is available through the Internet, instructors need to adopt a variety of strategies to enhance learners’ problem-solving abilities, and to enable them to learn how to harness and apply the knowledge.

#### The Future Research Target Can Be Extended Downward and Expanded to Different Education Levels

The circular inquiry learning method was effective in terms of helping the design students in this study improve the completeness of their project designs, and the students affirmed that this learning mode helped them to improve their confidence in inquiry. Therefore, the “QC/ADEAC inquiry model” and “QD/ODEAC inquiry model” can be used as an effective teaching tool, and it is suggested that the future research targets can be extended and expanded to different educational levels, in addition to the university students in this study.

### Limitations and Further Study

Because the teaching experiment of this study was conducted for 9 months, only highly motivated participants were invited to participate in this study in order to avoid sample attrition, which led to the problem of a small number of participants. Therefore, in future studies, the number of participants can be significantly increased to verify whether the concepts proposed in this study can be supported and inferred to different professional project designs.

Inquiry-based learning has been shown to be an effective way to increase students’ motivation in STEM subjects and increase their understanding of science concepts. Nevertheless, teachers do not often use inquiry-based learning in the classroom due to different (perceived) barriers (Silm et al., 2017). Despite researchers’ positive belief in inquiry-based learning, teachers tend to be less willing to implement inquiry-based learning in their courses, and claim that the implementation of inquiry-based learning is fraught with difficulties (Ramnarain and Hlatswayo, 2018). Therefore, future research could be conducted with higher education teachers to examine their perceptions and difficulties related to the implementation of inquiry-based learning courses in order to suggest specific teaching strategies for improvement.

In addition, inquiry-based learning and hands-on work are often combined with STEAM education concepts, which are considered to be more conducive to enhancing learners’ creative expression and hands-on skills. In the field of design, the concept of STEAM is also gaining importance. Therefore, exploring different types of STEAM education approaches will help to expand the understanding of the benefits of curriculum and instruction in design education. For example, the “C” in C-STEAM education is an abbreviation for culture, a goal-oriented concept that focuses on cultural transmission as the main educational goal (Huo et al., 2020). C-STEAM, with its strong traditional cultural characteristics, is considered to be conducive to the implementation of localized interdisciplinary education, and culture is an important part of design education. However, there is still limited understanding of the effects of different STEAM approaches applied in design education. Therefore, this study suggests that in future research, different STEAM methods can be combined with inquiry and practical work to compare their effects.

In addition, Xenofontos et al. (2020) suggested that appropriate instruction provided by the teacher during inquiry-based learning improved student learning outcomes more than uninstructed or minimally instructed inquiry-based learning for the same course material. However, the number of instructional sessions was not considered in this study, so it is not possible to understand the effect of different instructional intensities on student learning outcomes in the inquiry models presented in this study. Therefore, the effect of instructional intensity on inquiry-based learning can be investigated through multiple experimental group designs in subsequent studies. This study only explored the students’ inquiry and practical experiences, but did not use the students’ practical work as a basis for evaluation. Therefore, it is expected that future research will establish and develop a set of criteria for assessing students’ work, and allow teachers and students to complete them, so that a more comprehensive multiple assessment of students’ learning effectiveness can be implemented in the future, and besides, as seen in the previous literature, the implementation of inquiry-based learning can be combined with the use of educational technologies (e.g., apps, websites) in different ways. However, in this study, other educational technologies were not integrated, so the effects of technology-assisted inquiry-based learning on students’ cognition, emotions, knowledge, and skills can be explored in the future.

## Data Availability Statement

The raw data supporting the conclusions of this article will be made available by the authors, without undue reservation.

## Ethics Statement

Ethical review and approval was not required for the study on human participants in accordance with the local legislation and institutional requirements. The patients/participants provided their written informed consent to participate in this study.

## Author Contributions

J-YF and J-HY: concept and design and drafting of the manuscript, acquisition of data, and statistical analysis. J-YF: critical revision of the manuscript. Both authors contributed to the article and approved the submitted version.

## Conflict of Interest

The authors declare that the research was conducted in the absence of any commercial or financial relationships that could be construed as a potential conflict of interest.

## Publisher’s Note

All claims expressed in this article are solely those of the authors and do not necessarily represent those of their affiliated organizations, or those of the publisher, the editors and the reviewers. Any product that may be evaluated in this article, or claim that may be made by its manufacturer, is not guaranteed or endorsed by the publisher.

## References

[B1] Aditomo A. Goodyear P. Bliuc A. M. Ellis R. A. (2013). Inquiry-based learning in higher education: principal forms, educational objectives, and disciplinary variations. *Stud. High. Educ.* 38 1239–1258. 10.1080/03075079.2011.616584

[B2] Akbari O. Sahibzada J. (2020). Students’ self-confidence and its impacts on their learning process. *Am. Int. J. Soc. Sci. Res.* 5 1–15. 10.46281/aijssr.v5i1.462

[B3] Baars M. Wijnia L. Paas F. (2017). The association between motivation, affect, and self-regulated learning when solving problems. *Front. Psychol.* 8:1346. 10.3389/fpsyg.2017.01346 28848467 PMC5550677

[B4] Borovay L. A. Shore B. M. Caccese C. Yang E. Hua O. (2019). Flow, achievement level, and inquiry-based learning. *J. Adv. Acad.* 30 74–106. 10.1177/1932202X18809659

[B5] Bressler D. M. Shane Tutwiler M. Bodzin A. M. (2021). Promoting student flow and interest in a science learning game: a design-based research study of school scene investigators. *Educ. Technol. Res. Dev.* 69 2789–2811. 10.1007/s11423-021-10039-y

[B6] Bulunuz M. Jarrett O. S. Martin-Hansen L. (2012). Level of inquiry as motivator in an inquiry methods course for preservice elementary teachers. *Sch. Sci. Math.* 112 330–339. 10.1111/j.1949-8594.2012.00153.x

[B7] Cairns D. (2019). Investigating the relationship between instructional practices and science achievement in an inquiry-based learning environment. *Int. J. Sci. Educ.* 41 2113–2135. 10.1080/09500693.2019.1660927

[B8] Cairns D. Areepattamannil S. (2019). Exploring the relations of inquiry-based teaching to science achievement and dispositions in 54 countries. *Res. Sci. Educ.* 49 1–23. 10.1007/s11165-017-9639-x

[B9] Chen S. C. Yang S. J. Hsiao C. C. (2016). Exploring student perceptions, learning outcome and gender differences in a flipped mathematics course. *Br. J. Educ. Technol.* 47 1096–1112. 10.1111/bjet.12278

[B10] Chu S. K. W. Tse S. K. Loh E. K. Y. Chow K. (2011). Collaborative inquiry project-based learning: effects on reading ability and interests. *Libr. Inf. Sci. Res.* 33 236–243. 10.1016/j.lisr.2010.09.008

[B11] Clayton K. E. Blumberg F. C. Anthony J. A. (2018). Linkages between course status, perceived course value, and students’ preference for traditional versus non-traditional learning environments. *Comput. Educ.* 125 175–181. 10.1016/j.compedu.2018.06.002

[B12] Dlačić J. Arslanagić M. Kadić-Maglajlić S. Marković S. Raspor S. (2014). Exploring perceived service quality, perceived value, and repurchase intention in higher education using structural equation modelling. *Total Qual. Manag. Bus. Excell.* 25 141–157. 10.1080/14783363.2013.824713

[B13] Dubey S. Dubey A. K. (2017). Promotion of higher order of cognition in undergraduate medical students using case-based approach. *J. Educ. Health Promot.* 6:75. 10.4103/jehp.jehp_39_17 PMC5561721 28852665

[B14] Eckerlein N. Roth A. Engelschalk T. Steuer G. Schmitz B. Dresel M. (2019). The role of motivational regulation in exam preparation: results from a standardized diary study. *Front. Psychol*. 10:81. 10.3389/fpsyg.2019.00081 30804828 PMC6370677

[B15] Fan J. Y. Ye J. H. (2022). A study on teaching and learning model of project design course in a vocational and technological college and university. *Second. Educ.* 72 73–92. 10.6249/SE.202112_72(4).0030

[B16] Floyd K. S. Harrington S. J. Santiago J. (2009). The effect of engagement and perceived course value on deep and surface learning strategies. *Informing Sci. Int. J. Emerg. Transdiscipline* 12 181–190. 10.28945/435

[B17] Hair J. F. Black W. C. Babin B. J. Anderson R. E. Tatham R. L. (2019). *Multivariate data analysis*, 8th Edn. Boston: Cengage.

[B18] Hidi S. Renninger K. A. (2006). The four-phase model of interest development. *Educ. Psychol.* 41 111–127. 10.1207/s15326985ep4102_4

[B19] Hong J. C. Chen M. Y. Wong A. Hsu T. F. Peng C. C. (2012). Developing physics concepts through hands-on problem solving: a perspective on a technological project design. *Int. J. Technol. Des. Educ.* 22 473–487. 10.1007/s10798-011-9163-7

[B20] Hong J. C. Hsiao H. S. Chen P. H. Lu C. C. Tai K. H. Tsai C. R. (2021). Critical attitude and ability associated with students’ self-confidence and attitude toward “Predict-observe-explain” online science inquiry learning. *Comput. Educ.* 166:104172. 10.1016/j.compedu.2021.104172

[B21] Hong J. C. Hwang M. Y. Tai K. H. Tsai C. R. (2017). An exploration of students’ science learning interest related to their cognitive anxiety, cognitive load, self-confidence and learning progress using inquiry-based learning with an iPad. *Res. Sci. Educ.* 47 1193–1212. 10.1007/s11165-016-9541-y

[B22] Hong J. C. Tsai C. R. Hsiao H. S. Chen P. H. Chu K. C. Gu J. (2019). The effect of the “Prediction-observation-quiz-explanation” inquiry-based e-learning model on flow experience in green energy learning. *Comput. Educ.* 133 127–138. 10.1016/j.compedu.2019.01.009

[B23] Hong J. C. Ye J. H. Ho Y. J. Ho H. Y. (2020). Developing inquiry and hands-on learning model to guide STEAM lesson planning for kindergarten children. *J. Balt. Sci. Educ.* 19 908–922. 10.33225/jbse/20.19.908

[B24] Hong J. C. Yu K. C. Chen M. Y. (2011). Collaborative learning in technological project design. *Int. J. Technol. Des. Educ.* 21 335–347. 10.1007/s10798-010-9123-7

[B25] Hong J.-C. Hwang M.-Y. Liu M.-C. Ho H.-Y. Chen Y.-L. (2014). Using a “prediction-observation-explanation” inquiry model to enhance student interest and intention to continue science learning predicted by their Internet cognitive failure. *Comput. Educ.* 72 110–120. 10.1016/j.compedu.2013.10.00

[B26] Hsiao H. S. Hong J. C. Chen P. H. Lu C. C. Chen S. Y. (2017). A five-stage prediction-observation-explanation inquiry-based learning model to improve students’ learning performance in science courses. *Eurasia J. Math. Sci. Technol. Educ.* 13 3393–3416. 10.12973/eurasia.2017.00735a

[B27] Hui E. C. M. Bao H. (2013). The logic behind conflicts in land acquisitions in contemporary China: a framework based upon game theory. *Land Use Policy* 30 373–380. 10.1016/j.landusepol.2012.04.001

[B28] Huo L. Zhan Z. Mai Z. Yao X. Zheng Y. (2020). “A case study on C-STEAM education: Investigating the effects of students’ STEAM literacy and cultural inheritance literacy,” in *Technology in education. Innovations for online teaching and learning*, eds Lee L. K. Hou U L. Wang F. L. Cheung S. K. S. Au O. Li K. C. (Singapore: Springer), 3–12. 10.1007/978-981-33-4594-2_1

[B29] Jerrim J. Oliver M. Sims S. (2020). The relationship between inquiry-based teaching and students’ achievement. New evidence from a longitudinal PISA study in England. *Learn. Instr.* 10.1016/j.learninstruc.2020.101310

[B30] Johnson C. W. (1986). A more rigorous quasi-experimental alternative to the one-group pretest-posttest design. *Educ. Psychol. Meas.* 46 585–591. 10.1177/0013164486463011

[B31] Kapur M. (2008). Productive failure. *Cogn. Instr.* 26 379–424. 10.1080/07370000802212669

[B32] Kim Y. Steiner P. (2016). Quasi-experimental designs for causal inference. *Educ. Psychol.* 51 395–405. 10.1080/00461520.2016.1207177 30100637 PMC6086368

[B33] Knafo A. Schwartz S. H. (2004). Identity formation and parent-child value congruence in adolescence. *Br. J. Dev. Psychol.* 22 439–458. 10.1348/0261510041552765

[B34] Kori K. (2021). “Inquiry-based learning in higher education,” in *Technology supported active learning*, eds Vaz de Carvalho C. Bauters M. (Berlin: Springer), 59–74. 10.1007/978-981-16-2082-9_4

[B35] Krapp A. (2002). Structural and dynamic aspects of interest development: theoretical considerations from an ontogenetic perspective. *Learn. Instr.* 12 383–409. 10.1016/S0959-4752(01)00011-1

[B36] Levine M. D. (2009). “Differences in Learning and Neurodevelopmental Function in School-Age Children,” in *Developmental-Behavioral Pediatrics (Fourth Edition)*, eds Carey W. B. Crocker A. C. Coleman W. L. Elias E. R. Feldman H. M. (Amsterdam: Elsevier Inc), 535–546. 10.1016/B978-1-4160-3370-7.00055-9

[B37] Liem A. D. Lau S. Nie Y. (2008). The role of self-efficacy, task value, and achievement goals in predicting learning strategies, task disengagement, peer relationship, and achievement outcome. *Contemp. Educ. Psychol.* 33 486–512. 10.1016/j.cedpsych.2007.08.001

[B38] Liyadipita L. (2021). Self confidence and the cognitive styles among the secondary school students in Sri Lanka. *Kelaniya J. Manag.* 10:49. 10.4038/kjm.v10i0.7683

[B39] Maclellan E. (2014). How might teachers enable learner self-confidence? A review study. *Educ. Rev.* 66 59–74. 10.1080/00131911.2013.768601

[B40] Mansour N. (2015). Science teachers’ views and stereotypes of religion, scientists and scientific research: a call for scientist-science teacher partnerships to promote inquiry-based learning. *Int. J. Sci. Educ.* 37 1767–1794. 10.1080/09500693.2015.1049575

[B41] Mieg H. A. (2019). *Inquiry-based learning-Undergraduate research.* Berlin: Springer.

[B42] Mills N. Moulton S. T. (2017). Students’ and instructors’ perceived value of language and content curricular goals. *Foreign Lang. Ann.* 50 717–733. 10.1111/flan.12303

[B43] Mountrakis G. Triantakonstantis D. (2012). Inquiry-based learning in remote sensing: a space balloon educational experiment. *J. Geogr. High. Educ.* 36 385–401. 10.1080/03098265.2011.638707

[B44] Murphy C. Abu-Tineh A. Calder N. Mansour N. (2021). Teachers and students’ views prior to introducing inquiry-based learning in Qatari science and mathematics classrooms. *Teach. Teach. Educ.* 104:103367. 10.1016/j.tate.2021.103367

[B45] Muukkonen H. Lakkala M. Hallarainen K. (2005). Technology-mediation and tutoring: how do they shape progressive inquiry discourse? *J. Lean. Sci.* 14 527–565. 10.1207/s15327809jls1404_3

[B46] Newton X. A. Tonelli E. P. Jr. (2020). Building undergraduate STEM majors’ capacity for delivering inquiry-based mathematics and science lessons: an exploratory evaluation study. *Stud. Educ. Eval.* 64:100833. 10.1016/j.stueduc.2019.100833

[B47] Nguyen H. B. N. Hong J. C. Chen M. L. Ye J. N. Tsai C. R. (2021). Relationship between students’ hands-on making self-efficacy, perceived value, cooperative attitude and competition preparedness in joining an iSTEAM contest. *Res. Sci. Technol. Educ.* 1–20. 10.1080/02635143.2021.1895100

[B48] Özgüngör S. (2010). Identifying dimensions of students’ ratings that best predict students’ self efficacy, course value and satisfaction. *Eurasian J. Educ. Res.* 38 146–163.

[B49] Pedaste M. Mäeots M. Siiman L. A. De Jong T. Van Riesen S. A. Kamp E. T. (2015). Phases of inquiry-based learning: definitions and the inquiry cycle. *Educ. Res. Rev.* 14 47–61. 10.1016/j.edurev.2015.02.003

[B50] Pekrun R. Goetz T. Frenzel A. C. Barchfeld P. Perry R. P. (2011). Measuring emotions in students’ learning and performance: the Achievement Emotions Questionnaire (AEQ). *Contemp. Educ. Psychol.* 36 36–48. 10.1016/j.cedpsych.2010.10.002

[B51] Perry P. (2011). Concept analysis: confidence/self-confidence. *Nurs. Forum* 46 218–230. 10.1111/j.1744-6198.2011.00230.x 22029765

[B52] Ramnarain U. Hlatswayo M. (2018). Teacher beliefs and attitudes about inquiry-based learning in a rural school district in South Africa. *S. Afr. J. Educ.* 38:1413. 10.15700/saje.v38n1a1431

[B53] Safitri R. E. Widjajanti D. B. (2019). The effect of inquiry in scientific learning on students’ self-confidence. *J. Phys. Conf. Ser.* 1157:042073. 10.1088/1742-6596/1157/4/042073

[B54] Sara M. F. Maria T. (2013). Changes in interest and affect during a difficult reading task: relationships with perceived difficulty and reading fluency. *Learn. Instr.* 27 11–20. 10.1016/j.learninstruc.2013.02.001

[B55] Schrader C. Seufert T. Zander S. (2021). Learning from instructional videos: learner gender does matter; speaker gender does not. *Front. Psychol.* 12:655720. 10.3389/fpsyg.2021.655720 34122240 PMC8194702

[B56] Schwartz S. H. (1994). Are there universal aspects in the structure and contents of human values? *J. Soc. Issues* 50 19–45. 10.1111/j.1540-4560.1994.tb01196.x

[B57] Seitamaa-Hakkarainen P. Raunio A. M. Raami A. Muukkonen H. Hakkarainen K. (2001). Computer support for collaborative designing. *Int. J. Technol. Des. Educ.* 11 181–202. 10.1023/A:1011277030755

[B58] Sheldrake R. (2016). Confidence as motivational expressions of interest, utility, and other influences: exploring under-confidence and over-confidence in science students at secondary school. *Int. J. Educ. Res.* 76 50–65. 10.1016/j.ijer.2015.12.001

[B59] Silm G. Tiitsaar K. Pedaste M. Zacharia Z. C. Papaevripidou M. (2017). Teachers’ readiness to use inquiry-based learning: an investigation of teachers’ sense of efficacy and attitudes toward inquiry-based learning. *Sci. Educ. Int.* 28 315–325.

[B60] Stajkovic A. D. (2006). Development of a core confidence-higher order construct. *J. Appl. Psychol.* 91 1208–1224. 10.1037/0021-9010.91.6.1208 17100479

[B61] Suarez A. Specht M. Prinsen F. Kalz M. Ternier S. (2018). A review of the types of mobile activities in mobile inquiry-based learning. *Comput. Educ.* 118 38–55. 10.1016/j.compedu.2017.11.004

[B62] Teig N. Scherer R. Nilsen T. (2018). More isn’t always better: the curvilinear relationship between inquiry-based teaching and student achievement in science. *Learn. Instr.* 56 20–29. 10.1016/j.learninstruc.2018.02.006

[B63] Tretter T. R. Jones M. G. (2003). Relationships between inquiry-based teaching and physical science standardized test scores. *Sch. Sci. Math.* 103 345–350. 10.1111/j.1949-8594.2003.tb18211.x

[B64] Tseng C. H. Tuan H. L. Chin C. C. (2013). How to help teachers develop inquiry teaching: perspectives from experienced science teachers. *Res. Sci. Educ.* 43 809–825. 10.1007/s11165-012-9292-3

[B65] Wilhelm J. Sherrod S. Walters K. (2008). Project-based learning environments: challenging preservice teachers to act in the moment. *J. Educ. Res.* 101 220–233. 10.3200/joer.101.4.220-233

[B66] Xenofontos N. A. Hovardas T. Zacharia Z. C. de Jong T. (2020). Inquiry-based learning and retrospective action: problematizing student work in a computer-supported learning environment. *J. Comput. Assist. Learn.* 36 12–28.

[B67] Yang X. Zhang M. Kong L. Wang Q. Hong J. C. (2021). The effects of scientific self-efficacy and cognitive anxiety on science engagement with the “question-observation-doing-explanation” model during school disruption in COVID-19 pandemic. *J. Sci. Educ. Technol.* 30 380–393. 10.1007/s10956-020-09877-x 33169057 PMC7641485

[B68] Ye J. H. Wang C. M. Fan J. Y. Wu Y. F. Ye J. N. (2020). The association of hands-on making attitude, course interest, and continuous intention to participate in courses related to leather goods. *Taiwan Text. Res. J.* 30 64–72. 10.6439/TTRJ.202004_30(2).0008

[B69] Yin R. K. (2003). Designing case studies. *Qual. Res. Methods* 5 359–386.

[B70] Zheng L. (2021). *Data-driven design for computer-supported collaborative learning.* Singapore: Springer, 10.1007/978-981-16-1718-8_5

